# Recruitment of Mcm10 to Sites of Replication Initiation Requires Direct Binding to the Minichromosome Maintenance (MCM) Complex*

**DOI:** 10.1074/jbc.M115.707802

**Published:** 2015-12-30

**Authors:** Max E. Douglas, John F. X. Diffley

**Affiliations:** From The Francis Crick Institute, Clare Hall Laboratory, South Mimms, Hertfordshire EN6 3LD, United Kingdom

**Keywords:** DNA helicase, DNA polymerase, DNA replication, DNA synthesis, protein-DNA interaction, mcm10, replication origin

## Abstract

Mcm10 is required for the initiation of eukaryotic DNA replication and contributes in some unknown way to the activation of the Cdc45-MCM-GINS (CMG) helicase. How Mcm10 is localized to sites of replication initiation is unclear, as current models indicate that direct binding to minichromosome maintenance (MCM) plays a role, but the details and functional importance of this interaction have not been determined. Here, we show that purified Mcm10 can bind both DNA-bound double hexamers and soluble single hexamers of MCM. The binding of Mcm10 to MCM requires the Mcm10 C terminus. Moreover, the binding site for Mcm10 on MCM includes the Mcm2 and Mcm6 subunits and overlaps that for the loading factor Cdt1. Whether Mcm10 recruitment to replication origins depends on CMG helicase assembly has been unclear. We show that Mcm10 recruitment occurs via two modes: low affinity recruitment in the absence of CMG assembly (“G_1_-like”) and high affinity recruitment when CMG assembly takes place (“S-phase-like”). Mcm10 that cannot bind directly to MCM is defective in both modes of recruitment and is unable to support DNA replication. These findings indicate that Mcm10 is localized to replication initiation sites by directly binding MCM through the Mcm10 C terminus.

## Introduction

DNA replication in eukaryotes initiates from multiple sites, called replication origins. Replication initiation takes place in two steps: origin licensing, where DNA is made competent for replication, and origin firing, where DNA unwinding and synthesis begin (reviewed in Ref. 1). The primary events in origin licensing and firing center on loading and activating the core of the replicative helicase, the hexameric Mcm2–7 complex. During G_1_-phase, single hexamers of MCM^2^ are loaded onto DNA as inactive double hexamers (2, 3). In the ensuing S-phase, firing factors, including the protein kinases cyclin-dependent kinase (CDK) and Dbf4-dependent kinase (DDK), promote the recruitment of Cdc45 and GINS to loaded MCM, leading to assembly of the Cdc45-MCM-GINS (CMG) complex. The CMG complex is the replicative helicase that unwinds DNA at the replication fork (4, 5).

Mcm10 was identified in the same genetic screen as subunits of Mcm2–7 (6), but does not share sequence homology with them (7). Mcm10 is required for replication initiation (7, 8), but dispensable for MCM loading (2, 9), indicating that it plays a specific role in origin firing. Indeed, although CMG assembly can occur without Mcm10, DNA replication and the recruitment of replication protein A (RPA) to replication origins require Mcm10 (8, 10–13), suggesting that it acts at or downstream of helicase assembly. Despite this, the essential function of Mcm10 is unknown. The timing of Mcm10 recruitment to MCM has been controversial: Mcm10 has been shown to bind specifically to double hexamers of MCM in budding yeast cells during G_1_-phase (8), whereas the recruitment of Mcm10 to loaded MCM in yeast extracts was not detected until the two S-phase protein kinases, CDK and DDK, had acted (10). Thus far there has been no thorough analysis of Mcm10 recruitment to MCM in its different states with purified proteins. We have reconstituted Mcm10 recruitment *in vitro*. We show that Mcm10 can bind directly to MCM at a site that is composed of Mcm2 and Mcm6 and that overlaps the site for Cdt1. We find that the C terminus of Mcm10 is essential for direct binding to MCM and show that Mcm10 recruitment takes place via two modes, which we term “G_1_-like” and “S-phase-like.” Both modes of recruitment depend on the Mcm10 C terminus, indicating that the recruitment of Mcm10 to sites of replication initiation depends on direct binding to the MCM complex.

## Experimental Procedures

### 

#### 

##### Yeast Strains

All yeast strains are based on W303 and were constructed and manipulated by standard genetic techniques; details are listed in Table 1. The C-terminal 100 amino acids of an endogenous copy of MCM10 were deleted to form yMD16 by transformation of diploid w303 with PCR products generated using pBP83 (14). An endogenous copy of RAD9 was deleted in yMD16 by transformation with PCR products generated using pFA6a-natNT2 (15).

**TABLE 1:** ***Saccharomyces cerevisiae* strains**

Strain name	Genotype	Reference
yMD7	*MAT***a** *ade2-1 ura3-1 his3-11,15 trp1-1 leu2-3,112 can1-100 bar1::Hyg*	This study
	*pep4::KanMX*	
	*MCM6::MCM6-3* × *FLAG (Nat-NT2)*	
yMD8	*MAT***a** *ade2-1 ura3-1 his3-11,15 trp1-1 leu2-3,112 can1-100 bar1::Hyg*	This study
	*pep4::KanMX*	
	*MCM6::MCM6-3* × *FLAG (Nat-NT2)*	
	*his3::HIS3pRS303/CDT1,GAL4*	
	*trp1::TRP1pRS304/MCM4, MCM5 leu2::LEU2pRS305/MCM6^TC^, MCM7 ura3::URA3pRS306/MCM2, CBP-MCM3*	
yMD9	*MAT***a** *ade2-1 ura3-1 his3-11,15 trp1-1 leu2-3,112 can1-100*,	This study
	*cdc7-4, pep4:: Hyg*,	
	*MCM10::MCM10-3* × *FLAG (Nat-NT2)*	
	*his3::HIS3pRS303/SLD3-13MYC*,	
	*trp1::TRP1pRS304/SLD2, leu2::LEU2pRS305/SLD7, CDC45*,	
	*ura3::URA3pRS306/DPB11*	
yMD16	*MAT***a*/***α *ade2-1/ade2-1 ura3-1/ura3-1 his3-11,15/his3-11,15 trp1-1/trp1-1 leu2-3,112/leu2-3,112 can1-100/can1-100 MCM10^+^/mcm10Δ472-571 (Nat-NT2)*	This study
yMD17	*MAT***a*/***α *ade2-1/ade2-1 ura3-1/ura3-1 his3-11,15/his3-11,15 trp1-1/trp1-1 leu2-3,112/leu2-3,112 can1-100/can1-100 MCM10^+^/mcm10Δ472-571 (Nat-NT2) RAD9/rad9Δ::KanMX*	This study

Mutagenesis of MCM6 at the endogenous locus was done by integration of a cassette derived from pFA6a-natNT2 (15). A synthetic DNA fragment encoding amino acids 790–1017 of Mcm6 with tandem TEV-cleavable (tc) sites inserted after threonine 870 (see below) was cloned upstream of Nat-NT2. The resultant tc-MCM6(790–1017)-Nat-NT2 cassette was amplified by PCR and used to replace the 3′ region of an endogenous copy of MCM6 by transformation into diploid W303. Endogenous MCM6 and MCM10 were C-terminally tagged with 3×FLAG by transformation with PCR products generated using pBP83 (14).

##### DNA Templates

1-kb biotinylated linear ARS305 and 3.2-kb randomly biotinylated ARS1 circular templates were generated as described previously (13, 16). Biotinylated DNA was coupled to streptavidin-coated M-280 Dynabeads (Invitrogen) as described previously (16).

##### S-phase Extract

S-phase extract for protein recruitment assays was prepared as described previously (17), using extract from yeast strain yMD9, which is identical to strain yKO3 (17), except for the addition of a 3×FLAG tag on the C terminus of Mcm10. Where indicated, S-phase extract was depleted of Mcm10 by 1-h incubations four times at 4 °C with a 1:4 extract volume of anti-FLAG M2 magnetic beads (Sigma). Recruitment assays with extract were carried out as described previously (17), using 1-kb biotinylated ARS305 DNA templates.

##### Protein Purification

MCM complexes, MCM·Cdt1 complexes, ORC complexes, Cdc6, and individual MCM subunits were purified as in Ref. 14. tc-MCM was prepared as for MCM, except for an additional pulldown step with M2 anti-FLAG resin (Sigma) after purification to deplete endogenous 3×FLAG-tagged wild-type Mcm6. Purification of firing factors and execution of reconstituted replication assays in Figs. 3*e* and 4*d* was as in Ref. 13.

##### Protein Expression in Bacteria

All expression plasmids were transformed into BL21 DE3 Codon1 RIL cells (Stratagene). 4 liters of cells were grown at 25 °C to a density of *A*_600 nm_ = 0.6–0.8. Isopropyl-1-thio-β-d-galactopyranoside was added to 1 mm, and induction was carried out for 3–4 h at 25 °C. Cells were pelleted by centrifugation at 6,700 × *g* and washed once with phosphate-buffered saline before freezing and storage at −80 °C until use. All bacterial cells were lysed in the relevant lysis buffer supplemented with 0.5 mg/ml lysozyme (Sigma) for 30 min on ice, followed by sonication. Lysate was clarified by centrifugation at 23,600 × *g*.

##### Purification of MBP-Mcm6C

Bacterial cells transformed with pMD27 (MBP-Mcm6 789–1017, Table 2) were lysed in 25 mm Tris-HCl, pH 7.6, 10% glycerol, 0.05% Nonidet P-40-S, 1 m NaCl, 1 mm DTT, protease inhibitors (Roche Applied Science) (buffer J, 1 m NaCl + protease inhibitors). After clarification, supernatant was mixed for 1 h at 4 °C with amylose resin (GE Healthcare) preequilibrated in lysis buffer. After extensive washing, bound protein was eluted with lysis buffer + 10 mm maltose. Peak fractions were pooled and dialyzed against buffer J + 150 mm NaCl, before loading onto a MonoQ column preequilibrated in the same buffer. Protein was eluted over a gradient from 150 to 1000 mm NaCl. Peak fractions were pooled and snap-frozen. Maltose-binding protein (MBP) beads were prepared fresh from MBP-expressing bacterial lysate and washed extensively with buffer J + 1 m NaCl before use.

**TABLE 2:** **Plasmids generated in this study**

Name	Cloning vector	Insert	Generation of insert	5′ site	3′ site
pBP6	pET28a	*MCM10*	PCR (*S. cerevisiae* W303 genomic DNA template)	BamHI	BamHI
pMD84	pET28a	*MCM10* Δ*473–571*	PCR (pBP6 DNA template)	BamHI	BamHI
pMD65	pCS2-MYC-FA3	*MCM10*	PCR (pBP6 DNA template)	FseI	XbaI
pMD72	pCS2-MYC-FA3	*MCM10* Δ*1–371*	PCR (pBP6 DNA template)	FseI	XbaI
pMD73	pCS2-MYC-FA3	*MCM10* Δ*1–471*	PCR (pBP6 DNA template)	FseI	XbaI
pMD74	pCS2-MYC-FA3	*MCM10* Δ*473–571*	PCR (pBP6 DNA template)	FseI	XbaI
pMD75	pCS2-MYC-FA3	*MCM10* Δ*373–571*	PCR (pBP6 DNA template)	FseI	XbaI
pMD27	pMAL-c2P	*MCM6 789–1017*	PCR (full-length Mcm6 DNA template)	BamHI	SalI
pMD31	pFA6a-kanMX6	*MCM6 790–1017 + TC site at Thr-870*	PCR (tcMcm6 DNA template)	BamHI	BamHI

##### Purification of Mcm10

Mcm10 (full length and ΔC) was purified as in Ref. 13 from bacterial cells transformed with pBP6 (full length) or pMD84 (ΔC). Peak fractions from the second Mono S column were pooled, concentrated, and applied to a Superdex 200 size exclusion column preequilibrated with 25 mm Tris-HCl, pH 7.6, 10% glycerol, 0.01% Nonidet P-40-S, 500 mm NaCl (buffer M + 500 mm NaCl). Peak fractions were pooled, concentrated, and dialyzed against 25 mm Hepes, pH 7.6, 10% glycerol, 0.01% Nonidet P-40-S, 100 mm potassium glutamate (buffer MCM + 100 mm potassium glutamate), and aliquots were snap-frozen for storage.

##### Single Hexamer Pulldown Assays with Purified Mcm10

Purified single hexamers of MCM were preincubated on ice with 5 mm ATPγS in a buffer containing 25 mm Hepes, pH 7.6, 5 mm magnesium acetate, 0.02% Nonidet P-40-S, 10% glycerol, and 100 mm potassium glutamate (buffer MCM +100 mm potassium glutamate), the potassium glutamate concentration was raised to 500 mm, and purified Mcm10 was added. After 15 min on ice, binding mixes were centrifuged at 16,000 × *g* for 10 min at 4 °C. To precipitate Mcm10, the supernatant was incubated for 1 h with Ni-NTA beads washed with buffer MCM +500 mm potassium glutamate, with occasional mixing. Beads were washed three times with buffer MCM +500 mm potassium glutamate, and bound proteins were separated by SDS-PAGE and visualized with InstantBlue protein stain (Expedeon).

##### Double Hexamer Binding Assays with Purified Mcm10

MCM was loaded onto 1-kb biotinylated AR305 DNA templates as described (2), washed twice with buffer MCM +300 mm potassium glutamate, and incubated for 10 min at 30 °C with purified Mcm10 in a binding mix that contained 25 mm Hepes, 10% glycerol, 5 mm magnesium acetate, 0.02% Nonidet P-40-S, 300 mm potassium glutamate, 1 mm DTT, 1 mm ATP, and 0.2 mg/ml poly(dIdC)·poly(dIdC). Binding mixes were washed twice with buffer MCM +300 mm potassium glutamate, and DNA was photocleaved off beads as described (14) and analyzed by SDS-PAGE and silver staining.

##### Subunit Pulldown Assays with Bacterially Expressed Mcm10

Anti-T7 antibody beads (Abcam) were pre-bound to bacterially expressed, purified Mcm10 in buffer MCM + 100 mm potassium glutamate and washed one time with buffer MCM + 500 mm potassium glutamate. Purified MCM subunits were diluted into buffer MCM + 500 mm potassium glutamate and incubated with bead-bound Mcm10, or anti-T7 antibody beads alone, for 1.5 h on ice with occasional mixing. Binding reactions were washed three times with buffer MCM + 500 mm potassium glutamate before bound proteins were separated by SDS-PAGE and visualized with InstantBlue protein stain (Expedeon).

##### Pulldown Assays with Reticulocyte Lysate-expressed Mcm10

100 ng of plasmid DNA was added to 20 μl of reticulocyte lysate (Promega) with 10 μCi of [^35^S]methionine (PerkinElmer) to a final volume of 25 μl. Protein was expressed for 1.5 h at 30 °C, 100 μl of buffer MCM + 500 mm potassium glutamate was added, and the sample was divided between 5 μl of amylose resin pre-bound to either MBP-Mcm6C or MBP. Reactions were incubated on ice for 1.5 h before washing three times with buffer MCM + 500 mm potassium glutamate. Bound proteins were separated by SDS-PAGE and visualized by autoradiography.

##### Mcm10 and Cdt1 Competition Assays

Stoichiometric MCM·Cdt1 complexes purified from budding yeast (14) were incubated in buffer MCM +500 mm potassium glutamate, with 2 mm ATPγS and varying amounts of Mcm10 as indicated, for 20 min on ice. MCM was precipitated with anti-FLAG M2 magnetic beads (Sigma) for 1.5 h on ice, washed three times with buffer MCM + 500 mm potassium glutamate, and eluted with the same buffer supplemented with 1 mg/ml FLAG peptide. Eluted proteins were TCA-precipitated, separated by SDS-PAGE, and visualized by Western blotting.

##### Construction of TEV-cleavable Mcm6

A synthetic DNA fragment encoding part of Mcm6 was made in which the following DNA sequence was inserted after threonine 870: GGTGGTTCTGGTGGTGGTTCTGGTGAAAACCTGTATTTTCAGGGCGGTGGTTCTGGTGAAAACCTGTATTTTCAGGGCGGTGGTTCTGGTGGTGGTTCTGGT. This inserts the following amino acid sequence between Thr-870 and Gly-871: GGSGGGSGENLYFQGGGSGENLYFQGGGSGGGSG. This synthetic DNA fragment was cloned into the relevant vector for use as described.

##### Cleavage of tc-MCM with Tobacco Etch Virus (TEV) Protease

For experiments where double hexamers of tc-MCM were cleaved with TEV, MCM was loaded onto DNA, washed twice with buffer MCM +100 mm potassium glutamate, and incubated in the same buffer supplemented with 2 mm DTT and 0.2 mg/ml TEV protease. After 30 min at 30 °C, the sample was washed two times with buffer MCM +300 mm potassium glutamate before further use. When loaded MCM was phosphorylated with DDK in combination with TEV cleavage (Fig. 3, *d* and *e*), MCM was incubated with DDK as standard for 15 min after loading before TEV was added to 0.2 mg/ml, and incubation continued for a further 30 min. When single hexamers of MCM were treated with TEV, soluble MCM complex was incubated for 90 min at 25 °C in buffer MCM + 100 mm potassium glutamate supplemented with 2 mm DTT and 0.2 mg/ml TEV protease.

##### Antibodies

Mcm7 was detected with anti-Mcm7 (yN-19, sc-6688, Santa Cruz Biotechnology). FLAG-tagged proteins were detected with anti-FLAG M2 (Sigma). C-terminally HA-tagged tc-Mcm6 was detected with anti-HA (16B12, MMS-101R-200, Cambridge Bioscience). Polyclonal antibody against Cdc45 was as described (17). Mcm10 was detected via an N-terminal His tag with anti-His antibody (631212, Clontech). Cdt1 was detected with anti-Cdt1 antibody as described (18). Psf1 antibodies were a gift from K. Labib.

## Results

### 

#### 

##### Mcm10 Directly Binds MCM via Mcm2 and the Mcm6 C Terminus

**FIGURE 1.**
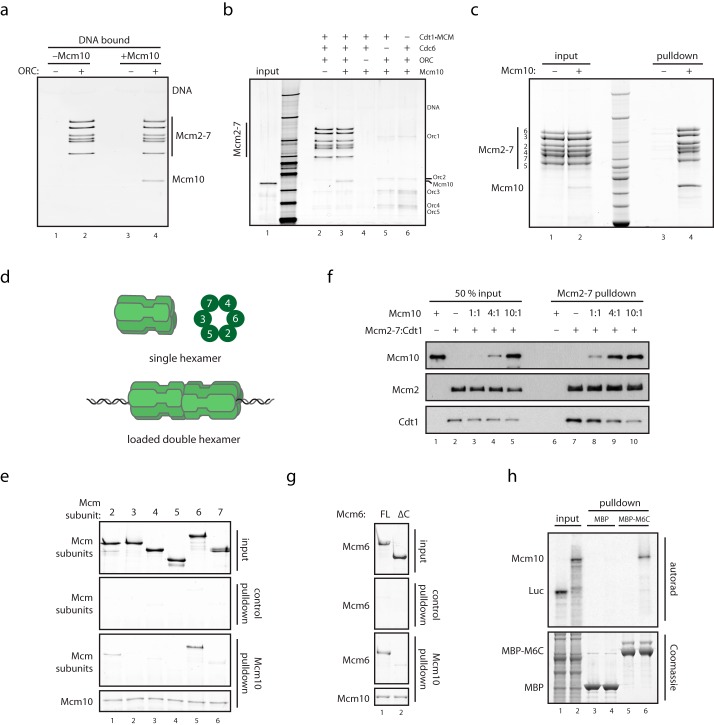
**Mcm10 binds directly to MCM via Mcm2 and Mcm6.** *a*, purified MCM was loaded onto DNA with purified loading factors and washed with high salt to remove ORC and Cdc6, and DNA-bound double hexamers were mixed with Mcm10 and analyzed for Mcm10 recruitment by SDS-PAGE and silver stain. *b*, MCM loading reactions were carried out as described (14), with individual loading factors excluded as indicated. Reactions were washed with low salt buffer, mixed with Mcm10, and washed, and DNA-bound proteins were analyzed by SDS-PAGE and silver stain. *c*, purified Mcm10 containing an N-terminal His tag was mixed with purified single hexamers of MCM. The sample was bound to Ni-NTA resin, which was washed and analyzed by SDS-PAGE and InstantBlue stain. *d*, schematic showing soluble MCM single hexamers, MCM double hexamers loaded onto DNA, and the order of subunits within the MCM ring. *e*, subunits of MCM were purified after overexpression in bacteria and mixed with anti-T7 resin bound to N-terminally T7-tagged Mcm10. Resin was washed, and bound proteins were analyzed by SDS-PAGE and InstantBlue stain. *f*, purified Mcm10 was mixed with a stoichiometric complex of MCM·Cdt1; MCM was immunoprecipitated with anti-FLAG resin via a 3×FLAG tag on Mcm3. Resin was washed, and bound proteins were analyzed by SDS-PAGE and Western blotting. *g*, full-length (*FL*) Mcm6 and Mcm6(1–871) purified after overexpression in bacteria were mixed with anti-T7 resin bound to N-terminally T7-tagged Mcm10. Resin was washed, and bound proteins were analyzed by SDS-PAGE and InstantBlue stain. *h*, MBP and MBP-Mcm6(789–1017) bound to glutathione-Sepharose resin were mixed with rabbit reticulocyte lysate containing ^35^S-labeled Mcm10 or luciferase (*Luc*). Resin was washed, and bound proteins were separated by SDS-PAGE and analyzed with InstantBlue stain or autoradiography.

To begin to characterize the role of Mcm10 in initiation, we first examined its interaction with the MCM double hexamer loaded onto bead-bound DNA with purified ORC, Cdc6, and Cdt1. As shown in Fig. 1*a*, near-stoichiometric amounts of Mcm10 were recruited to beads containing high salt-washed MCM double hexamers. Mcm10 recruitment was dependent on ORC, Cdc6, and Cdt1·MCM (Fig. 1, *a* and *b*). To determine whether this interaction is specific for the DNA-bound double hexameric form of MCM, we next tested whether bead-bound Mcm10 could interact with soluble MCM hexamers. As shown in Fig. 1*c*, MCM hexamer was efficiently pulled down by beads containing Mcm10, but not by beads lacking Mcm10. This indicates that Mcm10 can bind directly to MCM, but does not discriminate between soluble or loaded forms of MCM *in vitro*.

MCM subunits assemble in a defined spatial order that defines different functional interfaces on the MCM ring (14, 19) (Fig. 1*d*). To determine which interface interacts with Mcm10, we carried out binding assays with bead-bound Mcm10 and individual soluble MCM subunits (14, 19). As shown in Fig. 1*e*, Mcm10 bound near-stoichiometric amounts of Mcm6 (Fig. 1*e*, *lane 5*) and also bound Mcm2 (Fig. 1*e*, *lane 1*), although less efficiently. Biochemical and structural studies have shown that Mcm2 and Mcm6 are immediate neighbors in the MCM ring. Mcm10 also bound Mcm7 weakly (Fig. 1*e*, *lane 6*). Because this binding was very weak and because Mcm7 is not adjacent to Mcm2 and Mcm6 in the MCM ring, this interaction was not investigated further.

In addition to binding Mcm10, Mcm2 and Mcm6 also bind the replication factor Cdt1 (2, 20, 21). To determine whether Mcm10 and Cdt1 bind overlapping sites on MCM, we incubated a preformed Cdt1·MCM complex with different amounts of Mcm10 and analyzed which proteins were retained by immunoprecipitated MCM. Fig. 1*f* shows that as the concentration of Mcm10 was increased, MCM bound more Mcm10 but less Cdt1 (Fig. 1*f*, *lanes 7–10*). Cdt1 and Mcm10 can therefore compete with one another for binding to MCM, indicating that the binding sites on MCM for Mcm10 and Cdt1 overlap.

Cdt1 interacts with Mcm6 through a conserved winged helix domain in the C terminus (Mcm6C) (21–23). Consistent with the competition experiment described above, Mcm6 lacking this domain (Mcm6ΔC) was unable to bind Mcm10 (Fig. 1*g*). Moreover, a fusion of the Mcm6 C-terminal domain to MBP bound Mcm10, whereas MBP by itself did not (Fig. 1*h*), consistent with a previous yeast two-hybrid study (8). Therefore, the C terminus of Mcm6 is both necessary and sufficient for Mcm6 to bind Mcm10. Taken together, these results indicate that Mcm10 directly binds MCM at a site which overlaps that for Cdt1 and is composed of Mcm2 and the C terminus of Mcm6. Mcm10, however, also binds double hexamers of MCM (Fig. 1*a*), whereas Cdt1 does not (2). This indicates that, although overlapping, the specific interface bound by Cdt1 and Mcm10 must be, at least in part, different. It is interesting to consider that release of Cdt1 during MCM loading (24) may be important to reveal the Mcm10 binding site in the double hexamer.

##### The C Terminus of Mcm10 Is Required for Binding to MCM

**FIGURE 2.**
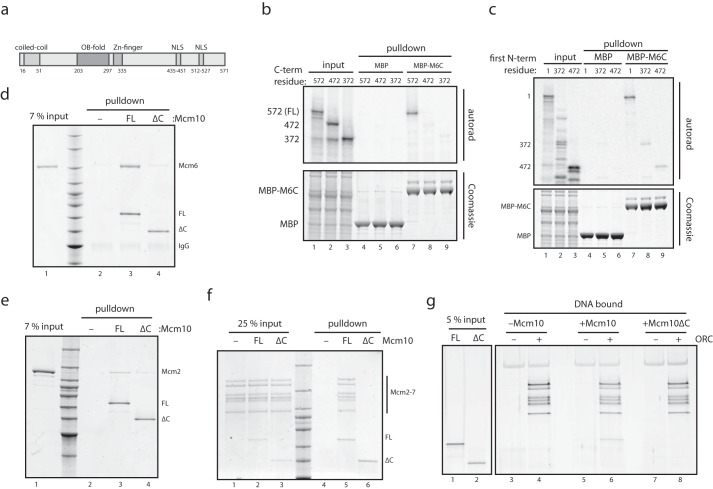
**The C terminus of Mcm10 is required for binding to the MCM complex.** *a*, schematic showing the domain structure of *S. cerevisiae* Mcm10. *NLS*, nuclear localization sequence. *b*, binding assays were carried out as in Fig. 1*h*, but with reticulocyte lysate containing ^35^S-labeled Mcm10 truncated from the C terminus as indicated. *FL*, full length. *c*, binding assays were carried out as in *b*, but with rabbit reticulocyte lysate containing ^35^S-labeled full-length Mcm10 or Mcm10 truncated from the N terminus as indicated. *d*, full-length Mcm10 and Mcm10(1–471) (Mcm10ΔC) were bound to anti-T7 resin and mixed with full-length untagged Mcm6. Resin was washed, and bound proteins were analyzed by SDS-PAGE and InstantBlue stain. *e*, the binding assay in *d* was repeated with full-length Mcm2 instead of Mcm6. *f*, full-length Mcm10 and Mcm10(1–471) (Mcm10ΔC) were mixed with single hexamers of MCM and then mixed with Ni-NTA resin, which was washed, and bound proteins were analyzed by SDS-PAGE and InstantBlue stain. *g*, purified MCM was loaded onto DNA with purified proteins, washed with high salt to remove ORC and Cdc6, and then DNA-bound double hexamers were mixed with full-length Mcm10 or Mcm10ΔC and washed, and bound proteins were analyzed by SDS-PAGE and silver stain.

Next, we determined which region of Mcm10 mediates binding to MCM. Mcm10 comprises an N-terminal coiled-coil (25), a central OB-fold and zinc finger domain (26), and two C-terminal nuclear localization motifs (Fig. 2*a*). In metazoans, the Mcm10 C terminus contains a winged helix domain (27), although this is not seen in the yeast protein. Truncation of 99 amino acids or more from the C terminus (Mcm10ΔC) prevented Mcm10 from binding to the C terminus of Mcm6 (Fig. 2*b*, *lanes 7–9*), whereas a polypeptide containing just the last 100 amino acids of Mcm10 was specifically retained by Mcm6C (Fig. 2*c*). This indicates that the C terminus of Mcm10 is necessary and sufficient for binding to the C terminus of Mcm6. We also found that Mcm10ΔC was unable to bind full-length Mcm6 (Fig. 2*d*), establishing that the C terminus of Mcm10 and the C terminus of Mcm6 are the only points of contact between these proteins. Yeast two-hybrid experiments with fruit fly Mcm10 have suggested that Mcm2 binds Mcm10 in part via the Mcm10 C terminus (28). Consistent with this, Fig. 2*e* shows that budding yeast Mcm10ΔC was defective in binding to Mcm2 (Fig. 2*e*). Finally, we tested whether the C-terminal 100 amino acids of Mcm10 are necessary to bind to single and double hexamers of MCM. As shown in Fig. 2, *f* and *g*, Mcm10ΔC failed to interact with either single hexamers of MCM in solution (Fig. 2*f*, *lanes 5* and *6*) or double hexamers of MCM loaded onto DNA (Fig. 2*g*, *lanes 6* and *8*). These experiments show that the C terminus of Mcm10, which is required for binding to Mcm2 and Mcm6 (Fig. 2, *d* and *e*), is essential for Mcm10 to interact with the MCM complex.

##### Mcm6C Is Not Required for Double Hexamer Maintenance or Mcm10 Recruitment

**FIGURE 3.**
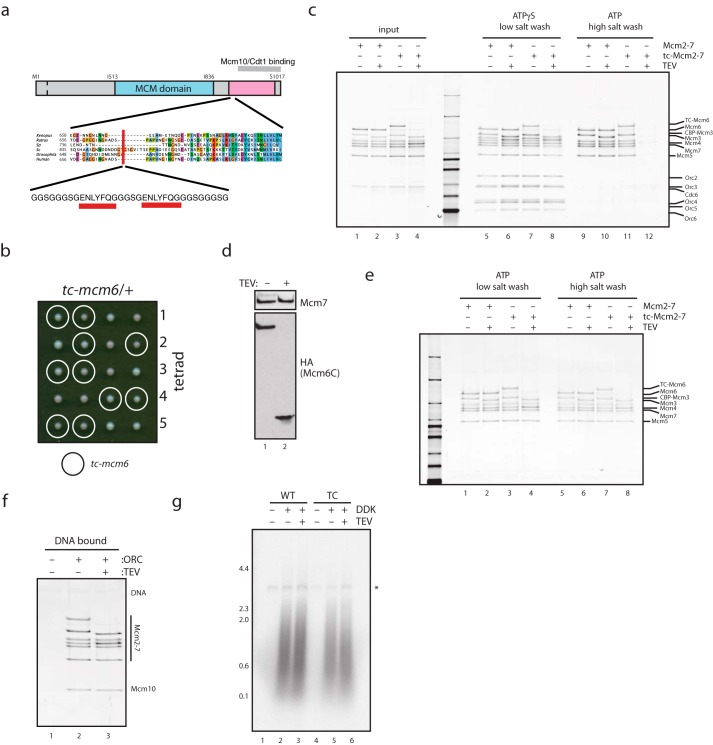
**The C terminus of Mcm6 is not required for Mcm10 recruitment, or DNA replication initiation.** *a*, schematic of the tc-Mcm6 construct developed in this study. *b*, diploid budding yeast cells containing one copy each of wild-type and TEV-cleavable MCM6 were sporulated, and tetrads were analyzed for growth, indicating that TEV cleavage site insertion has no effect on viability. *c*, purified MCM·Cdt1 or tc-MCM·Cdt1 heptamers (which contain tc-Mcm6) were incubated with TEV protease and used in double hexamer assembly assays in the presence of ATP or the poorly hydrolyzed ATP analogue ATPγS. DNA-bound proteins were analyzed by SDS-PAGE and silver stain. Although all complexes were recruited to DNA under low salt conditions in the presence ATPγS (*lanes 5–8*), TEV cleavage of tc-Mcm6 prevented high salt (0.5 m NaCl)-resistant MCM loading (*lane 12*). Note that CBP-Mcm3 shifts in mobility ±TEV protease due to a TEV cleavage site between CBP and Mcm3. *d*, Mcm2–7 containing tc-Mcm6 was loaded onto DNA with purified proteins and incubated with TEV protease as indicated. DNA-bound proteins were separated by SDS-PAGE and analyzed by Western blotting. *e*, MCM and tc-MCM were loaded onto DNA, washed with high salt buffer, cut with TEV protease as indicated, and washed with low salt (0.3 m potassium glutamate) or high salt buffer as indicated. *f*, MCM and tc-MCM were loaded onto DNA, washed with high salt, cut with TEV protease as indicated, washed with low salt, and mixed with purified Mcm10. DNA was washed, and bound proteins were analyzed by SDS-PAGE and silver stain. *g*, MCM and tc-MCM were loaded onto DNA, cut with TEV protease as indicated, and used in a DNA replication system reconstituted with purified proteins (13). Radiolabeled replication products were separated by denaturing alkaline agarose electrophoresis and visualized by autoradiography. The *asterisk* marks nicked plasmid products that are labeled in a replication-independent manner.

As described above, the C terminus of Mcm6 interacts with Cdt1 and is therefore required for MCM loading (21–23). Consequently, we cannot examine the significance of the Mcm10-Mcm6C interaction by simply deleting Mcm6C. To determine whether the binding of Mcm10 to Mcm6C plays an essential role after loading, we developed a modified version of MCM in which Mcm6 contains a cleavage site for the TEV protease such that Mcm6C can be specifically removed by cleavage after MCM loading (Fig. 3*a*). This allowed us to separate the essential function of Mcm6C in MCM loading from any role it may play subsequently in replication. tc-Mcm6 was functional (Fig. 3*b*), and efficiently loaded onto origin DNA by purified proteins (Fig. 3*c*, compare *lanes 9* and *11*). Cleavage of single hexamers of tc-MCM prior to loading prevented salt-stable association of MCM with DNA (a key property of MCM double hexamers (2), Fig. 3*c*, *lanes 11* and *12*), thus confirming that Mcm6C is required for MCM double hexamer assembly. Similar results have been obtained with a simple deletion of Mcm6C (21).

With this system in hand, we next asked whether Mcm6C is required to maintain double hexamers after loading. Loaded double hexamers containing tc-Mcm6 were cleaved efficiently and the C terminus of Mcm6 was removed after washing (Fig. 3, *d* and *e*, *lanes 3* and *4*,), but this had no effect on the resistance of DNA-bound MCM to challenge with high salt (Fig. 3*e*, *lanes 7* and *8*). Therefore, the C terminus of Mcm6 does not appreciably affect the stability of MCM double hexamers once formed.

Next, we addressed whether Mcm6C is required for the binding of Mcm10 to MCM, or for the function of Mcm10 in replication initiation. Fig. 3*f* shows that removal of the Mcm6 C terminus had no effect on the binding of Mcm10 to MCM double hexamers. Furthermore the efficiency of replication, as well as the profile of replication products synthesized with purified proteins (13), was unaffected by the presence or absence of Mcm6C (Fig. 3*g*, compare *lanes 5* and *6*). The C terminus of Mcm6 is therefore not required for the binding of MCM to Mcm10 or for the replication initiation function of Mcm10. These data show that binding to Mcm6 is not required for Mcm10 to interact with MCM. This is very similar to Cdt1, which binds tightly to Mcm6C, but binds MCM hexamers lacking Mcm6C (21), and suggests that Mcm2, alone or in combination with Mcm6, mediates direct binding of both Cdt1 and Mcm10 to the MCM complex.

##### Mcm10 Is Recruited to Replication Origins via Two Modes

**FIGURE 4.**
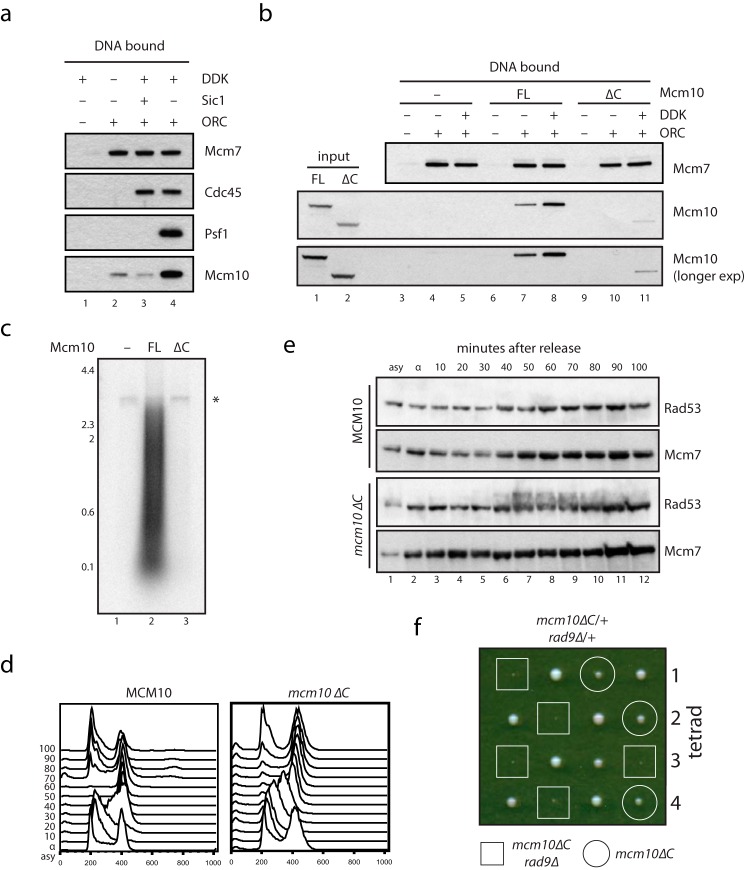
**Mcm10 is recruited via two modes that require direct binding to MCM.** *a*, replication complex assembly was analyzed with a previously described extract-based assay (17). Cdc45 required MCM loading and DDK activity for recruitment to DNA, whereas GINS required MCM loading and CDK and DDK activity. The recruitment of Mcm10 to DNA required MCM loading and was highest when CDK and DDK were active, but also occurred weakly without the activity of either kinase. *b*, to analyze the recruitment of Mcm10ΔC to replication initiation complexes, the replication complex assembly assay in *a* was carried out with extract depleted of endogenous Mcm10 and supplemented with buffer (*lanes 3–5*), full-length (*FL*) Mcm10 (*lanes 6–8*), or Mcm10ΔC (*lanes 9–11*). DNA-bound proteins were separated by SDS-PAGE and analyzed by Western blotting. Mcm10ΔC is severely defective for both G_1_-like (*lane 10*) and S-phase-like (*lane 11*) recruitment. *c*, MCM was loaded onto DNA and used in a DNA replication system reconstituted with purified proteins including Mcm10 or Mcm10ΔC (13). Radiolabeled replication products were separated by denaturing alkaline agarose electrophoresis and visualized by autoradiography. The *asterisk* marks nicked plasmid products that are labeled in a replication-independent manner. *d*, budding yeast cells containing full-length Mcm10 or Mcm10ΔC as indicated were released from an α-factor-induced block, and cells were collected at the time point indicated. DNA synthesis was followed by FACS analysis. *e*, extracts from the budding yeast cells analyzed in *d* were separated by SDS-PAGE and analyzed by Western blotting with antibodies raised against Rad53 or Mcm7 as indicated. Rad53 becomes activated toward the end of S-phase specifically in *mcm10*Δ*C* cells. *f*, one copy of Rad9 was deleted from diploid budding yeast cells heterozygous for *mcm10*Δ*C*, which were sporulated, and tetrads were analyzed for growth. *mcm10*Δ*C* and *rad9*Δ show a severe synthetic growth defect.

Whether Mcm10 recruitment happens before or after CMG assembly has been controversial (8–13, 29). To address this, we analyzed the recruitment of Mcm10 to replication initiation complexes in S-phase extracts that support DNA replication (17, 30). MCM loaded onto DNA with purified proteins was phosphorylated with DDK before the addition of S-phase extract from budding yeast cells (17). The recruitment of Cdc45 to MCM required DDK activity (Fig. 4*a*, *lanes 2* and *3*), whereas GINS recruitment required both DDK and CDK (Fig. 4*a*, *lane 4*), (17). We next analyzed Mcm10 recruitment. The recruitment of Mcm10 to DNA was strongest when MCM was loaded and both CDK and DDK were active (Fig. 4*a*, *lane 4*). However, a small amount of Mcm10 was also bound to MCM without DDK and CDK activity (Fig. 4*a*, *lanes 2* and *3*). This suggests that Mcm10 is recruited in two modes: with high affinity during or after CMG assembly, and with low affinity before CMG assembly has taken place.

We term these two modes of recruitment G_1_-like in the absence of CMG assembly (Fig. 4*a*, *lanes 2* and *3*), and S-phase-like when CMG assembly can take place (Fig. 4*a*, *lane 4*). We suggest that this difference in binding avidity may explain the disparate conclusions of previous studies.

##### Mcm10 Recruitment Requires Direct Binding to MCM

To ask whether both modes of recruitment are mediated by direct binding of Mcm10 to MCM, Mcm10 was depleted from an S-phase extract, and either nothing or full-length Mcm10 or Mcm10ΔC was added back. Although full-length Mcm10 recapitulated the recruitment pattern of endogenous Mcm10 (Fig, 4*b*, *lanes 7* and *8*), the recruitment of Mcm10ΔC was not observed in the absence of CDK, and was severely defective in the presence of CDK (Fig. 4*b*, *lanes 10* and *11*). We conclude that direct binding of Mcm10 to MCM is required for both G_1_-phase-like and S-phase-like modes of Mcm10 recruitment to sites of replication initiation.

##### Direct Binding of Mcm10 to MCM Is Required for DNA Replication

Mcm10 is essential for DNA replication initiation (10, 13). To test whether this function depends on direct binding to MCM, we examined whether Mcm10ΔC can support replication *in vitro* and *in vivo*. As shown in Fig. 4*c*, full-length Mcm10 supported robust replication of a DNA plasmid in the reconstituted replication system used in Fig. 3 (13). In contrast, synthesis was severely reduced in the presence of Mcm10ΔC. This shows that direct recruitment of Mcm10 by MCM is essential for normal levels of DNA replication to take place.

Budding yeast cells containing *mcm10*ΔC as the only copy of Mcm10 were viable but grew slowly (Fig. 4*f*, *circles*), and exhibited an extended S-phase in synchronous cell cultures (Fig. 4*d*). The checkpoint kinase Rad53 was also hyperphosphorylated late in S-phase in these Mcm10ΔC cells (Fig. 4*e*). The DNA damage response may repair replication errors in the face of inefficient replication initiation in *mcm10*Δ*C* cells. Indeed, deletion of the checkpoint mediator *RAD9* caused a severe growth defect with *mcm10*Δ*C* such that *mcm10*Δ*C*, *rad9*Δ cells were barely viable (Fig. 4*f*). We suggest that *mcm10*Δ*C* cells are viable because a small amount of residual S-phase-like Mcm10 recruitment can take place even when the C terminus has been deleted (Fig. 4*b*), although this is clearly not sufficient for replication *in vitro* (Fig. 4*c*). We conclude that direct binding of Mcm10 to MCM through the C terminus of Mcm10 is crucial for normal DNA replication.

## Discussion

**FIGURE 5.**
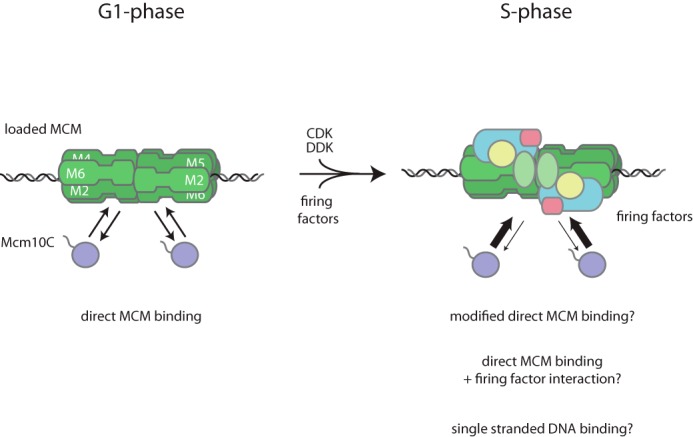
**A summary of Mcm10 recruitment based on the findings presented in this study.** Details are described under “Results.”

How Mcm10 is localized to replication origins was previously unclear. Here, we show that Mcm10 is recruited through two modes: low affinity or stability in the absence of CMG assembly (G_1_-like), and high affinity or stability when CMG assembly can take place (S-phase-like, Fig. 5). We find that both modes of recruitment depend on the direct binding of Mcm10 to MCM via the C terminus of Mcm10. G_1_-like recruitment could result in helicase activation in the presence of Cdc45 and GINS, which may in turn lead to stable S-phase-like recruitment due to the appearance of single-stranded DNA, to which Mcm10 could bind directly (26). Interestingly, disruption of the C terminus of fission yeast Mcm10 prevents DNA replication *in vivo* (31), suggesting that the recruitment mechanism we have uncovered here is conserved.

Mcm10 binds the MCM ring at an interface including Mcm2 and Mcm6 (Fig. 1). Binding to Mcm6 is not required for Mcm10 recruitment or function (Fig. 3), indicating that the interaction between Mcm10 and Mcm2 is likely to be important. Mcm10ΔC is weakened but not completely defective for binding to Mcm2, indicating that other parts of the protein must play a role. In *Drosophila*, the central region of Mcm10, which contains OB-fold and zinc finger motifs, has been suggested to bind Mcm2 (28), and in budding yeast, growth defects caused by mutations in this region are suppressed by mutations in Mcm2 (32). Interestingly, in a recent high-resolution structure of loaded MCM, some of these suppressor mutations map closely to a side channel between Mcm2 and Mcm6 that is a putative extrusion site for single-stranded DNA (33). Binding on this interface of the MCM ring may position Mcm10 in prime position to access any single-stranded DNA that is extruded during the replication initiation process.

In budding yeast extract, Mcm10 specifically associates with DNA-bound complexes containing MCM double hexamers, but not soluble single hexamers (8). Given that this is not the case with purified proteins (Fig. 1), we suggest that Cdt1, which binds single hexamers at a site overlapping that for Mcm10 (Fig. 1), might ensure that Mcm10 associates specifically with the loaded pool of MCM. It is interesting to speculate that this takes place during G_1_ to ensure that in the following S-phase, all CMG complexes that are assembled are also activated.

## Author Contributions

M. E. D. and J. F. X. D. conceived all experiments and wrote the paper; M. E. D. performed all experiments.

## Footnotes

2 The abbreviations used are:

MCM: minichromosome maintenance
CDK: cyclin-dependent kinase
DDK: Dbf4-dependent kinase
ORC: origin recognition complex
TEV: tobacco etch virus
tc: TEV-cleavable
MBP: maltose-binding protein
ATPγS: adenosine 5′-*O*-(thiotriphosphate)
Ni-NTA: nickel-nitrilotriacetic acid
CBP: calmodulin binding peptide.
